# Enhanced immunogenicity of a positively supercharged archaeon thioredoxin scaffold as a cell-penetrating antigen carrier for peptide vaccines

**DOI:** 10.3389/fimmu.2022.958123

**Published:** 2022-08-09

**Authors:** Davide Cavazzini, Gloria Spagnoli, Filipe Colaco Mariz, Filippo Reggiani, Stefano Maggi, Valentina Franceschi, Gaetano Donofrio, Martin Müller, Angelo Bolchi, Simone Ottonello

**Affiliations:** ^1^ Department of Chemistry, Life Sciences & Environmental Sustainability, University of Parma, Parma, Italy; ^2^ German Cancer Research Center (DKFZ), Tumorvirus-specific Vaccination Strategies (F035), Heidelberg, Germany; ^3^ Department of Veterinary Science, University of Parma, Parma, Italy; ^4^ Interdepartmental Center Biopharmanet-Tec, University of Parma, Parma, Italy

**Keywords:** antigen carrier, recombinant vaccine, peptide epitope, intracellular antigen delivery, epitope display, protein scaffold engineering, protein scaffold vaccine, protein DNA interaction

## Abstract

Polycationic resurfaced proteins hold great promise as cell-penetrating bioreagents but their use as carriers for the intracellular delivery of peptide immuno-epitopes has not thus far been explored. Here, we report on the construction and functional characterization of a positively supercharged derivative of *Pyrococcus furiosus* thioredoxin (*Pf*Trx), a thermally hyperstable protein we have previously validated as a peptide epitope display and immunogenicity enhancing scaffold. Genetic conversion of 13 selected amino acids to lysine residues conferred to *Pf*Trx a net charge of +21 (starting from the -1 charge of the wild-type protein), along with the ability to bind nucleic acids. In its unfused form, +21 *Pf*Trx was readily internalized by HeLa cells and displayed a predominantly cytosolic localization. A different intracellular distribution was observed for a +21 *Pf*Trx-eGFP fusion protein, which although still capable of cell penetration was predominantly localized within endosomes. A mixed cytosolic/endosomal partitioning was observed for a +21 *Pf*Trx derivative harboring three tandemly repeated copies of a previously validated HPV16-L2 (aa 20-38) B-cell epitope grafted to the display site of thioredoxin. Compared to its wild-type counterpart, the positively supercharged antigen induced a faster immune response and displayed an overall superior immunogenicity, including a substantial degree of self-adjuvancy. Altogether, the present data point to +21 *Pf*Trx as a promising novel carrier for intracellular antigen delivery and the construction of potentiated recombinant subunit vaccines.

## Introduction

Intracellular delivery of proteins is an important goal of current biomedical research, aimed at expanding the site of action and range of applications of protein-based therapeutic, prophylactic and diagnostic macromolecules. Different ways to achieve this goal have been proposed in recent years (1). These range from various kinds of nano-carriers (2), to the genetic grafting of positively charged peptide modules (designated as Cell Penetrating Peptides, CPP; or Protein Transduction Domains, PTD) (3–5) or amphipatic α-helixes (also known as Translocation Motifs, TLM) that promote protein internalization (6), to the use of positively supercharged (PSC) cell-penetrating proteins (1). The latter can be either naturally occurring proteins (7), or designed proteins bearing a net positive charge >1 per kDa of molecular mass (8, 9).

Construction of positively charged proteins can entail the targeted substitution of a cluster of surface-exposed, neutral or negatively charged amino acids with positively charged (Lys/Arg or, more rarely, His) residues (so called ‘arginine-grafting mutagenesis´) (3), or the replacement of all negatively charged or neutral, surface-exposed residues with positively charged amino acids (8, 9). A pioneering proof-of-concept demonstration of the latter approach, also known as ‘genetic resurfacing’, has been obtained through the systematic mutagenesis of the 29 amino acids exposed on the surface of green fluorescent protein (GFP), which, starting from a net charge of -7 (superfolder sfGFP variant), yielded multiple variants with a net charge of up to +48 (10).

In contrast to positive supercharging, negatively super-charged GFP was found to be unable to penetrate cells on its own (11), but became internalization-competent upon complexation with positively charged lipids (12). Positively supercharged GFP variants, instead, proved capable of autonomous cell penetration, both alone and with a terminally fused non-supercharged protein cargos, with a concentration-dependent increase in internalization and a sort of midpoint transition around a theoretical net positive charge of +21 (8, 13). Although a clear and generally applicable threshold for the ability of PSC proteins to enter mammalian cells has not been defined yet, a net positive charge at physiological pH/molecular mass ratio (Rcm) of at least +0.75/kDa seems to be required (8).

The best characterized polycationic resurfaced protein is +36 GFP, which has been shown to outperform various CPPs and PTMs as a transduction agent capable of promoting cell penetration of different genetically fused cargo proteins (13) as well as nucleic acids (11) into different cell types. In addition to cell penetration capacity, positive supercharging confers other properties to resurfaced GFP, such as an enhanced resistance against temperature- and chemical perturbation-induced aggregation (10).

Although successfully applied to a few other proteins in addition to GFP (e.g., streptavidin and GST), the generality of genetic resurfacing as a means to confer new properties to a protein without interfering with its natural function is still a matter of debate (1). Indeed, even structurally similar proteins have been found to differently respond to surface modification and many proteins of therapeutic interest did not withstand polycationic resurfacing (9). Accordingly, the use of a single or a few functionally validated, positively supercharged proteins as vehicles for the intracellular delivery of different cargos has been proposed as a potentially valuable strategy to overcome the uncertainties associated to *de novo* protein resurfacing (1).

PSC protein internalization has been shown to be energy-dependent and to rely on interaction with cell surface-associated, negatively charged sulfated proteoglycans and glycosaminoglycans. In keeping with endocytosis as the main entry pathway, most internalized PSC proteins –most notably +36 GFP fusion derivatives- at least initially appear inside cells as punctate endosomal foci (13, 14). Thus, a major problem following internalization is endosome escape and release of the PSC protein into the cytosol, which occurs to different extents with different PSC proteins. For example, at variance with the only partial escape observed with resurfaced +36 GFP as well as with its Arg-grafted derivative (9), the lack of an appreciable endosomal accumulation and a nearly complete release into the cytosol have been reported for polycationic resurfaced nanobodies (i.e., the antigen-binding domains of camelid-derived VHH antibodies) fused to wt-GFP (15).

Despite the above described limitations, a number of interesting proof-of-principle applications of PSC proteins (especially but not exclusively +36 GFP) have been reported in recent years. These range from the intracellular delivery of nucleic acids and proteins (11, 13), multiprotein assembly design (16, 17), including the fabrication of nanoencapsulated enzyme reactors (18, 19), to the construction of biosensor arrays (20) and the development of a high-performance split-GFP system (21).

Another important goal in the field of intracellular protein delivery is the development of recombinant subunit vaccines capable of penetrating professional Antigen Presenting Cells (e.g., dendritic cells) in order to be processed intracellularly and presented to T-cells by class I (cytotoxic CD8+ T-cells) or class II (helper CD4+ T-cells) Major Histocompatibility Complex (MHC) molecules. Two main approaches for intracellular antigen delivery have been pursued in recent years. One of them, mainly applied to MHC class I presentation, relies on the use of different types of micro/nanoparticles, including virus-like particles, polymersomes, hydrogels/microgels and liposomes (22, 23), and takes advantage of the phagocytic properties of dendritic cells or membrane fusion-mediated intracellular delivery. In the other approach, applied to both soluble and particulate antigens and to class I as well as to class II MHC presentation, various peptide motifs (CPPs, PTDs and TLMs) have been used to promote antigen internalization (6, 24–26). To our knowledge, only one case has been reported in which a polycationic resurfaced protein (+36 GFP) has been employed for the non-covalent adsorption and intracellular delivery of a human papillomavirus (HPV) E7-based immunogen in the form of a DNA vaccine (27).

To test the potential of PSC proteins as peptide vaccine carriers, we constructed and characterized a positively charged (+21) version of *Pyrococcus furiosus* thioredoxin (*P*fTrx), a small protein (100 aa) whose ability to act as an effective scaffold for the presentation of B-cell epitopes inserted into its active/display site has previously been documented (28–30). Genetic resurfacing was well tolerated by *Pf*Trx, which in different experimental settings displayed a robust cell penetration capacity, with a sizeable fraction of the protein present in the cytosol. A derivative of +21 *Pf*Trx harboring three tandemly repeated copies of a previously validated immune epitope from HPV minor capsid protein L2 genetically grafted into the display site of thioredoxin (28, 29, 31) was used as a proof-of-concept antigen for immunogenicity evaluation. Compared to its wild-type (wt) *Pf*Trx counterpart, the supercharged +21 *Pf*Trx-(HPV16-L2)_3x_ antigen induced a faster and stronger immune response even in the absence of an immune-adjuvant. Altogether, our data point to +21 *Pf*Trx as a promising and potentially general carrier protein for intracellular antigen delivery and the construction of potentiated recombinant peptide vaccines.

## Results

### Polycationic resurfacing of *P. furiosus* thioredoxin

**Figure 1:**
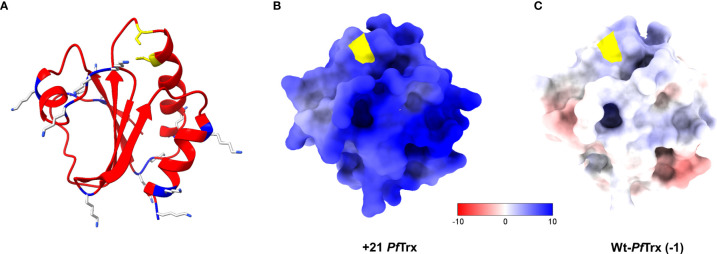
Structure and electrostatic surface potential of +21 *Pf*Trx. **(A)** Ribbon representation of the 3D structure of +21 *Pf*Trx predicted with AlphaFold2 (32). The 13 amino acids that have been replaced with Lys residues are shown as *light grey* and *blue* sticks; the two active/display site Cys residues are in *yellow*. The electrostatic surface potentials (ESP) of +21 *Pf*Trx and of its wild-type counterpart (net negative charge=-1) are illustrated in panels **(B, C)**, respectively; ESPs are represented in a false color scale ranging from −10 kT/e (*red*) to +10 kT/e (*blue*).

A positively supercharged derivative of *Pf*Trx was designed according to the rules outlined by the Liu laboratory (8). Specifically, using the predicted structure of *Pyrococcus* thioredoxin as a reference (28), selected surface-exposed, neutral or negatively charged amino acids not involved in intramolecular contacts were replaced by lysine residues (**Figure 1A**). Amino acids located within (or very close to) the two cysteine residues of the active site of thioredoxin that is used for peptide epitope grafting and display (shown in *yellow* in **Figure 1A**) as well as amino acid residues conserved within the expanded family of thioredoxin orthologs were similarly spared from lysine replacement. A total of 13 amino acids of a display site engineered version of *Pf*Trx (28) were thus converted to lysine residues (see **Figure S1A** for sequence details). Compared to wt-*Pf*Trx, which has a net charge of -1 and an Rcm of -0.08/kDa, the polycationically resurfaced *Pf*Trx derivative has a net charge of +21 and an Rcm value of 1.77/kDa (1.57/kDa taking into account the contribution of the 6xHis-tag). The electrostatic surface potentials of +21- and wt-*Pf*Trx are illustrated in **Figures 1B, C**.

**Figure 2:**
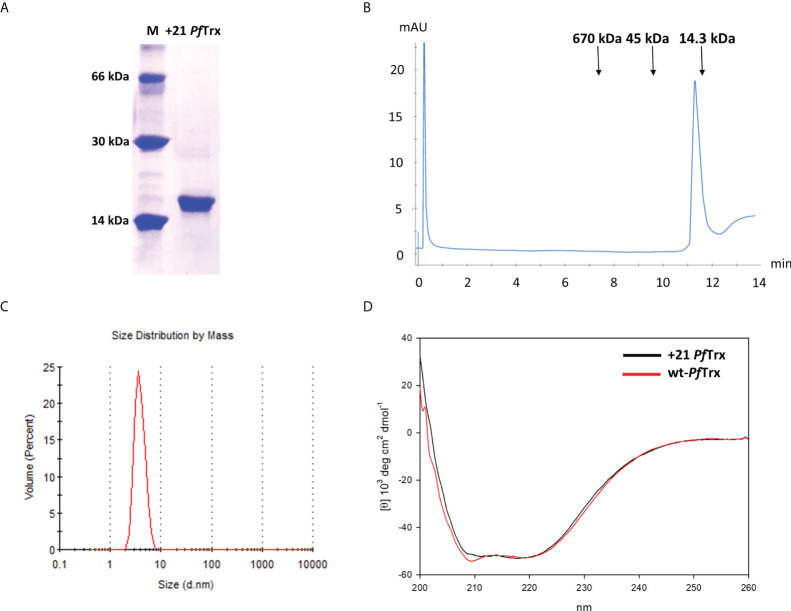
Biochemical characterization of +21 *Pf*Trx. **(A)** SDS-PAGE profile of the purified +21 *Pf*Trx protein; molecular mass markers (*M*; bovine serum albumin, carbonic anhydrase and lysozyme, from top to bottom are shown in the *left lane*). **(B)** Size exclusion chromatography analysis of the purified +21 *Pf*Trx protein performed on an analytical Superose 6 Increase 5/150 GL column; the sizes and elution positions of the molecular mass standards utilized for column calibration (thyroglobulin, ovalbumin and lysozyme, from *left to right*) are indicated. **(C)** Diameter size, monodispersed distribution of +21 *Pf*Trx measured by dynamic light scattering. **(D)** Comparative circular dichroism analysis of +21 *Pf*Trx (*black line*) and wt-*Pf*Trx (*red line*).

As shown in **Figure S1B**, His-tagged +21 *Pf*Trx was purified to homogeneity in two steps: an initial metal-affinity chromatography, in which imidazole-mediated elution was preceded by extensive washing with 2 M NaCl in order to remove nucleic acids and other cellular polyanions adsorbed to (or strongly interacting with) the PSC protein, followed by additional polishing on a cation exchange column. A highly purified product, with an apparent electrophoretic mobility under denaturing SDS-PAGE conditions slightly lower than expected (an anomalous migration behavior likely due to positive supercharging) was thus obtained (**Figure 2A**), with a final yield of approximately 6 mg of purified protein/liter of bacterial culture, which is about six-fold lower compared to that commonly obtained with wt-*Pf*Trx (28). Purified +21 *Pf*Trx eluted as a single peak from size exclusion chromatography (SEC) conducted under native conditions, with an apparent molecular mass (17 kDa) close to the expected size of the protein (14 kDa) (**Figure 2B**). A similar result in terms of structural homogeneity was obtained by Dynamic Light Scattering (**Figure 2C**), which yielded an estimated diameter of 4.18 nm, that is very close to the expected value for a monomeric protein and nearly identical to that measured under the same experimental conditions for lysozyme (14.6 kDa; estimated diameter of 4.16 nm).

Importantly, as revealed by circular dichroism (CD) analysis (**Figure 2D**), the alpha-helical content of +21 *Pf*Trx is essentially the same as that of wt-*Pf*Trx, indicating that polycationic resurfacing did not alter the secondary structure of the protein. Despite an overall structural conservation, however, +21*Pf*Trx was no longer recognized by an anti-wt *Pf*Trx antibody and a specific anti-supercharged *Pf*Tx monoclonal antibody (mAb) had to be developed for subsequent studies (see below). In addition, similar to the situation previously reported for positively supercharged GFP (10), a decreased thermal stability was observed for +21 *Pf*Trx. In particular, starting from the hyper-thermostable wt-*Pf*Trx protein [no change in ellipticity after a 1 h incubation at 100°C (28)], a thermal unfolding CD analysis revealed a progressive loss of ellipticity with an estimated midpoint transition around 61°C (**Figure S1C**).

We also verified the ability of positively supercharged *Pf*Trx to bind nucleic acids (**Figure S2**). This was documented by: i) the formation, visualized by Atomic Force Microscopy, of heterogeneously sized large particles (ranging from 200 to 500 nm in diameter) (**Figures S2A–C**) and a shift in electrophoretic mobility upon addition of M13 DNA (8 kbp) to +21- but not wt-*Pf*Trx (**Figures S2D**); and ii) the single-stranded DNA (64-mer oligonucleotide) concentration-dependent appearance of a void volume-eluting macromolecular complex detected by SEC with +21 *Pf*Trx (**Figure S2E**, *left-panel*) but not with wt *Pf*Trx (not shown), nor with a similarly sized (14.3 kDa) but less positively charged protein (net charge=+8; Rcm=0.55/kDa) such as lysozyme (**Figure S2E**, *right panel*).

### Cell penetration capacity of +21 PfTrx

**Figure 3:**
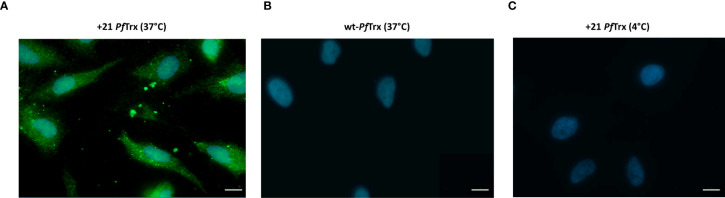
Immunofluorescence microscopy analysis of the cell penetration capacity of +21 *Pf*Trx. HeLa cells were incubated in the presence of 0.1 µM positively supercharged **(A)** or wild-type **(B)** *Pf*Trx in serum-free medium for 4 h at 37°C, then washed (3X) with 20 U/ml heparin in order to remove cell surface-adsorbed proteins. A representative image of a control incubation performed with +21 *Pf*Trx at 4°C is shown in panel **(C)**. Anti +21 *Pf*Trx **(A, C)** and anti-wt-*Pf*Trx **(B)** specific mAbs were used for protein detection (*green*); nuclei were stained with DAPI (*blue*) (see ‘Materials and Methods’ for details). Scale bars represent 10 µm.

Next, we used immunofluorescence microscopy along with an *ad hoc* developed anti-supercharged *Pf*Tx mAb, to investigate the cell penetration capacity of +21 *Pf*Trx which was added to HeLa cells at concentration of 0.1µM, in the lower range of those previously employed for similar studies on the cellular internalization of different PSC proteins, especially +36 GFP (13). As shown in **Figure 3A**, a fairly strong, cytosol-diffuse fluorescence signal was detected in HeLa cells incubated at 37°C with 0.1 µM +21 *Pf*Trx, whereas no fluorescence was observed in a parallel incubation carried out with the non-resurfaced wt-*Pf*Trx protein (**Figure 3B**). Similarly, no fluorescence signal was detected upon incubation with +21 *Pf*Trx at 4°C (**Figure 3C**), thus pointing to temperature-dependent endocytic internalization as the main uptake process. This mode of internalization is consistent with several results previously reported for other PSC proteins, including +36 GFP, which, however, has been mostly studied in a protein cargo-fused (or nucleic acid complex) form rather than in a free, unconjugated form and found to accumulate within endosomes, with only a partial release into the cytosol. The latter distribution differs from the predominantly cytosolic distribution of +21 *Pf*Trx, which closely resembles the preferential cytosolic localization of polycationic resurfaced nanobodies (15).

**Figure 4:**
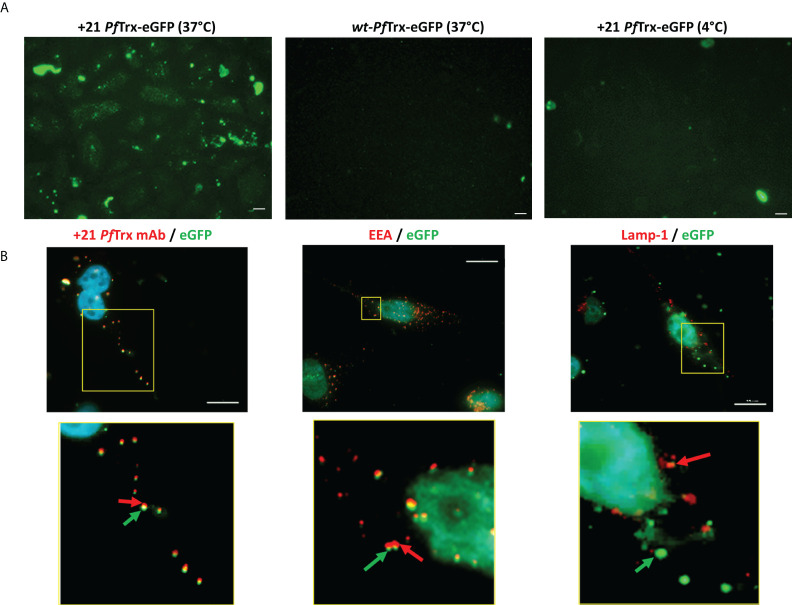
Cellular internalization of +21 *Pf*Trx-eGFP analyzed by fluorescence microscopy. **(A)** HeLa cells were incubated for 4 hours at 37°C in the presence of 0.5 µM +21 *Pf*Trx-eGFP (*left panel*) or wt-*Pf*Trx-eGFP (*middle panel*), or with +21 *Pf*Trx-eGFP at 4°C (*right panel*). After extensive washing with heparin (see above), internalized eGFP fluorescence was directly visualized by fluorescence microscopy. **(B)** Immunofluorescence analysis of the cell internalization and subcellular localization of +21 *Pf*Trx-eGFP in HeLa cells incubated as in **(A)**. Images on the *left*, based on intrinsic eGFP fluorescence (*green*) and on the fluorescence signal associated with the anti +21 *Pf*Trx antibody (*red*), highlight the colocalization of +21 *Pf*Trx and the eGFP cargo. Images shown in the middle panels, based on intrinsic eGFP fluorescence (*green*) and on the *red* fluorescence derived from an anti-Early Endosomal Antigen (EEA) antibody, document the endosome localization of +21 PfTrx-eGFP and its association with endosomal foci. Right-side images, in which the *green* fluorescent spots identify eGFP and the *red* fluorescent spots are generated by an anti-lysosomal-associated membrane protein 1 (LAMP-1) antibody, indicate the partial lysosomal localization of +21 *Pf*Trx-eGFP. Images shown in the bottom panels are magnified views of the *yellow-boxed* areas shown in the corresponding upper panels. Nuclei were stained with DAPI (*blue*) in all images. Scale bars represent 10 µm.

To further investigate these different internalization behaviors, we constructed a fusion derivative of +21 *Pf*Trx bearing a molecule of eGFP genetically attached to its N-terminus (see **Figure S3**). The resulting +21 *Pf*Trx-eGFP fusion protein was incubated with HeLa cells, which were then analyzed by fluorescence microscopy. As shown in **Figure 4A** (*left panel*), a fairly intense fluorescence signal was clearly visible in HeLa cells incubated for 4 h at 37°C in the presence of a fixed concentration (0.5 µM) of +21 *Pf*Trx-eGFP and extensively washed with a 20 U/ml heparin solution prior to visualization. In contrast, only very little background fluorescence was detected with the wt-*Pf*Trx-eGFP fusion protein (*middle panel*) as well as upon incubation of at 4°C (*right panel*), which again points to endocytosis as the main cellular process responsible for internalization. An immunofluorescence microscopy approach was then used to gain more detailed insight into cell penetration by +21 *Pf*Trx-eGFP. As shown in **Figure 4B**, +21 *Pf*Trx colocalized with eGFP within intracellular punctate structures (*left panel*) that were co-stained by an antibody directed against the Early Endosome Antigen 1 (EEA1) marker **(**Pearson’s correlation coefficient ~0.8; *middle panel*). In contrast, a well distinct and spatially separated staining was observed upon visualization of +21-*Pf*Trx-fused eGFP and the Lysosome Associated Lysosome Marker 1 (LAMP-1) protein (Pearson’s correlation coefficient ~0.3; *right panel*).

Besides confirming a prevalent endosomal localization of internalized +21 *Pf*Trx-eGFP, yet distinguishing it from a purely degradative lysosomal sequestration, these data also attest to the structural integrity of supercharged *Pf*Trx and its eGFP cargo. Moreover, a comparison of the intracellular fluorescence detected with the unconjugated and the eGFP-fused version of +21 *Pf*Trx (*cf*. **Figures 3**, **4**) indicates that the latter protein not only tends to be entrapped within endosomes, but also displays an overall reduced internalization capacity. Importantly, and in keeping with previous results indicating the lack of any cytotoxicity of +36 GFP and its fusion derivatives (13), no morphological alteration was observed in HeLa cells exposed to +21 *Pf*Trx or +21 *Pf*Trx-eGFP even at the highest concentrations (not shown). Moreover, a less than 5% loss of cell viability was detected with MTT assays conducted on HeLa cells treated with +21 *Pf*Trx concentrations (1.25-10 µM) 10- to 100-fold higher than those utilized for internalization experiments (**Figure S4**; see also **Figure 3** and ‘Materials and Methods’ for details). A 10% to 30% reduction of cell viability, more marked with wt-*Pf*Trx than with +21 *Pf*Trx, was only observed at the highest (20 µM) protein concentration (**Figure S4**).

### Construction and assessment of the cellular internalization capacity of the +21 PfTrx-(HPV16-L2)_3x_ antigen

Based on prior results indicating the superior immunogenicity of cell penetrating antigens (6, 23–26), we then constructed a +21 *Pf*Trx derivative bearing three tandemly repeated copies of a 19 aa peptide from HPV16 minor capsid protein L2 (major cross-neutralization epitope aa 20-38) as a homotypic multiepitope previously shown to elicit robust anti-HPV neutralizing antibody responses when fused to thioredoxin (28, 29, 31).

Compared to the empty form of PSC thioredoxin, the +21 *Pf*Trx-(HPV16-L2)_3x_ derivative has a molecular mass of 20.5 kDa, a theoretical net charge of +25 and an Rcm of 1.3/kDa (1.22 considering the contribution of the 6xHis-tag) (see **Figures S5A, B**). Similar to the empty form of positively supercharged thioredoxin, +21 *Pf*Trx-(HPV16-L2)_3x,_ which was also purified to homogeneity with a two-step metal-affinity/cation exchange chromatographic procedure, displays an SDS-PAGE electrophoretic mobility slightly lower than expected (**Figure S5C**). In keeping with the results obtained with +21-*Pf*Trx, the CD spectra of the +21 *Pf*Trx and the *Pf*Trx HPV16-L2 multiepitope-containing proteins are nearly identical (**Figure S5D**), suggesting the absence of any appreciable secondary structure alteration induced on the (HPV16-L2)_3x_ insert by supercharged +21 *Pf*Trx scaffold. The thermal stabilities of the two forms of +21 *Pf*Trx revealed by CD analysis (*cf*. **Figure S1C** and **Figure S5E**) were nearly identical, with an estimated midpoint transition at approximately 60°C for both forms of the protein, compared to the absence of any appreciable transition even at 85°C previously measured for *Pf*Trx-L2(20-38)_3x_ (28).

**Figure 5:**
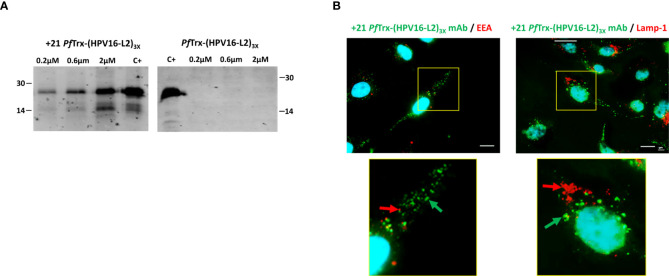
Cell penetration capacity of the +21 *Pf*Trx-(HPV16-L2)_3x_ antigen. **(A)** HeLa cells were incubated for 4 hrs. at 37°C in the presence of increasing concentrations of +21 *Pf*Trx-(HPV16-L2)_3x_ or wt-*Pf*Trx-(HPV16-L2)_3x_ as indicated, and washed three times with 20 U/ml heparin. After cell lysis, the resulting soluble extracts were separately subjected to SDS-PAGE fractionation and the two *Pf*Trx-(HPV16-L2)_3x_ proteins were detected by immunoblotting using an anti-L2 monoclonal antibody (see ‘Materials and Methods’ for details). +21 *Pf*Trx-(HPV16-L2)_3x_ (*left-side* gel) and wt-*Pf*Trx-(HPV16-L2)_3x_ (*right-side* gel) were used as controls (C+) for the corresponding gel fractionation/immunoblot experiments; the migration positions and sizes (kDa) of two molecular weight markers used for gel calibration are shown on the *left-side* and the *right-side* of each gel image, respectively. **(B)** Immunofluorescence microscopy analysis of cell internalization and subcellular distribution of the +21 *Pf*Trx-(HPV16-L2)_3x_ antigen. Following incubation (4 hrs. at 37°C) of +21 *Pf*Trx-(HPV16-L2)_3x_ (0.1 µM) with Hela cells, and heparin-washing as in **(A)**, the fusion protein was visualized with the use of a mAb directed against positively supercharged *Pf*Trx (*green-fluorescent* spots, marked by *green arrows*). An additional antibody directed against Early Endosomal Antigen (EEA) was used for the experiments shown in the *left-side* panels to detect endosomes, which were visualized as *red-fluorescent* spots (marked by *red arrows*). An antibody directed against lysosomal-associated membrane protein 1 (LAMP-1) was used for the experiments shown in the *right-side* panels to detect lysosomes, which were visualized as *red-fluorescent* spots (marked by *red arrows*); also in this set of experiments, the +21 *Pf*Trx-(HPV16-L2)_3x_ protein was detected with a +21 *Pf*Trx-specific mAb and visualized as *green-fluorescent* spots (marked by *green arrows*). Images shown in the bottom panels are magnified views of the *yellow-boxed* areas shown in the corresponding upper panels. Nuclei were stained with DAPI (*blue*) in all images. Scale bars represent 10 µm.

We initially used an immunoblotting approach to investigate the ability of +21 *Pf*Trx-(HPV16-L2)_3x_ to penetrate HeLa cells. As shown in **Figure 5A**, upon incubation with increasing amounts of PSC thioredoxin bearing the HPV16-L2 (aa 20-38) multiepitope, followed by extensive washing with a heparin-containing solution and detection with an anti-HPV-L2 mAb, a dose-dependent signal increase was observed with +21 *Pf*Trx-(HPV16-L2)_3x_ but not with the corresponding wt-*Pf*Trx-(HPV16-L2)_3x_ protein. The cell penetration capacity of +21 *Pf*Trx-(HPV16-L2)_3x_ was further verified by immunofluorescence microscopy analysis (**Figure 5B**) that confirmed the ability of the PSC antigen to be taken up by HeLa cells through an endosome-dependent process in a predominantly cytosolic form (diffuse or associated to non-endosomal punctate structures) (*left panel*), with only a modest accumulation within mature lysosomes (*right panel*) (see **Figure 5B** legend for the relevant Pearson’s correlation coefficient colocalization values).

Cumulative data reported in **Figure 5**, obtained with the use of anti-L2 and anti +21 *Pf*Trx mAbs, indicate that positive supercharging of the thioredoxin scaffold effectively promotes cellular internalization and cytosolic release of an intact, immunoreactive form of the HPV16-L2 multiepitope.

### Enhanced immunogenicity of the cell-permeant +21 PfTrx(HPV-L2)_3x_ vaccine prototype

Following-up to the above data, we wished to find out whether a potentially more effective MHC class II presentation resulting from cytosolic delivery of the +21 *Pf*Trx-(HPV16-L2)_3x_ antigen may influence its immunogenicity properties. To do so, assuming that addition of an immune-adjuvant was still required to achieve an effective humoral immune response, we initially tested the effect on cellular internalization of different human-use approved adjuvants (AddaVax, an MF-59-like adjuvant; aluminum hydroxide; aluminum phosphate; cyclic di-AMP; and the CpG oligodeoxynucleotide) as well as their chemical compatibility with +21 *Pf*Trx-(HPV16-L2)_3x_. When mixed with the PSC antigen, both aluminum salts as well as CpG ODN formed insoluble material, which accumulated on the surface of HeLa cells in a heparin unwashable form (not shown). AddaVax and cyclic di-AMP (CDA), instead, appeared to be fully compatible with +21 *Pf*Trx-(HPV16-L2)_3x_ and did not interfere with its internalization (**Figure S6**).

**Figure 6:**
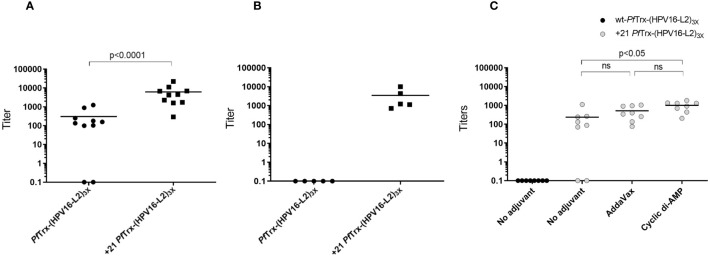
Immunogenic performance of the +21 *Pf*Trx-(HPV16-L2)_3x_ antigen. **(A)** Neutralizing antibody titers against HPV16 induced in mice immunized intramuscularly with the AddaVax-adjuvanted +21 *Pf*Trx- and wt-*Pf*Trx-based *Pf*Trx(HPV16-L2)_3x_ antigens, measured with the pseudovirion-based L1 neutralization assay. The results shown are the neutralization titers of individual immune-sera from mice (10 animals/group) immunized with +21 *Pf*Trx- or wt-*Pf*Trx-based antigens as indicated; the geometric means of the titers for each group are indicated by horizontal lines. **(B)** Same as **(A)** but with the use of a shorter immunization protocol (i.e., immune sera collected after two rather than three boost injections) applied to two groups of five animals each. **(C)** HPV16 neutralizing antibody titers of immune-sera from mice (8 animals/group) vaccinated with the unadjuvanted wt-*Pf*Trx-(HPV16-L2)_3x_ antigen (*black circles*), or with the AddaVax (50% v/v) or the cyclic di-AMP (7.5 μg/dose)-adjuvanted +21 *Pf*Trx-(HPV16-L2)_3x_ antigen (*grey circles*) as indicated. Statistical significance (p < 0.05) was evaluated with the non-parametric Mann-Whitney-Wilcoxon test, with the exclusion of intergroup comparisons in which neutralization titers for one of the two groups were close to zero.

We then performed a mouse immunization experiment using a standard vaccination protocol (one priming followed by three boost intramuscular injections; see ‘Materials and Methods’ for details) in order to compare the anti-HPV antibody responses elicited by AddaVax-adjuvanted +21 *Pf*Trx-(HPV16-L2)_3x_ with those induced by the same HPV16-L2 multiepitope grafted to wt-*Pf*Trx. As shown in **Figure 6A**, significantly higher anti-HPV16 neutralizing antibody titers were induced by +21 *Pf*Trx-(HPV16-L2)_3x_ compared to the corresponding wt-*Pf*Trx-based antigen. Likely due to its markedly altered surface charge and/or cell penetration capacity the PSC antigen, compared to wt-*Pf*Trx-(HPV16-L2)_3x_, induced a Th1-skewed immune response with a well detectable production of IgG2a immunoglobulins (**Figure S7**).

To further evaluate the apparent superiority of the PSC antigen, we repeated the same comparison using a shorter, more stringent immunization protocol only involving two boost injections. Also under these suboptimal immunization conditions, +21 *Pf*Trx-(HPV16-L2)_3x_ outperformed the corresponding non-supercharged antigen (**Figure 6B**).

Finally, we followed-up to the above results, by asking whether an adjuvant was absolutely required to promote +21 *Pf*Trx-(HPV16-L2)_3x_ immunogenicity. To address this question, we compared the immune responses induced by +21 *Pf*Trx-(HPV16-L2)_3x_ alone or supplemented with AddaVax or CDA. As shown in **Figure 6C**, slightly reduced but still well detectable HPV neutralizing antibody titers (as high as 90% of the maximum value measured in this experiment with only two non-responders) were obtained with the +21 *Pf*Trx-(HPV16-L2)_3x_ antigen in the absence of an exogenously supplied adjuvant. Under the same conditions, no measurable neutralizing antibody response was induced by the non-adjuvanted wt-*Pf*Trx antigen. This suggests that *Pf*Trx supercharging and the ensuing cellular internalization of the +21 *Pf*Trx-(HPV16-L2)_3x_ antigen not only speeds-up antibody production and/or affinity maturation, but also potentiates antigen recognition thus conferring a substantial degree of self-adjuvanticity.

## Discussion

The major bottleneck that limits full exploitation of protein-based therapeutics beyond extracellular or cell surface-exposed targets, is intracellular delivery. In this work, we explored polycationic resurfacing, a promising approach for the intracellular delivery of proteins, as a tool for potentiating peptide vaccine immunogenicity. We specifically focused on a positively charged variant of *P. furiosus* thioredoxin —a thermally stable protein whose immune epitope presentation capacity and lack of immunological cross-reactivity with animal thioredoxins have previously been documented (28–30)— as a macromolecular scaffold displaying on its surface a HPV16 minor capsid protein L2 homotypic multiepitope. Our data indicate that in addition to its role in the presentation of conformationally constrained linear epitopes, PSC thioredoxin is capable of promoting cellular internalization of internally grafted peptide epitopes. This new function critically depends on the net positive charge of resurfaced *Pf*Trx. In fact, very little cellular uptake was observed with a *Pf*Trx derivative bearing a +13 (data not shown), rather than a +21 net positive charge, which has previously been identified as the cellular internalization midpoint in a graded series of increasingly supercharged GFP variants (8, 13). However, charge density, rather than the net positive charge *per se*, is thought to be most critical for cellular internalization and an Rcm value of approximately 0.75/kDa has been experimentally determined as a sort of minimum requirement for effective cell penetration. With regard to this parameter, +21 *Pf*Trx, with an Rcm value (1.75) similar to that of +48 GFP (1.6), yet higher than that of +36 GFP (1.2) and of resurfaced nanobodies (1.0), falls within the upper range of the Rcm values of various natural PSC proteins whose internalization capacity has been experimentally evaluated (7). In keeping with its high positive charge and Rcm values, +21 *Pf*Trx was shown to be capable of binding single-stranded as well as double-stranded DNA molecules ranging in size from 64 to 64,000 nt.

The relative contribution of molecular size expansion and Rcm reduction to the drop in cell penetration capacity is yet unclear. Particularly with regard to a possible difference in cellular internalization capacity between terminal fusions such as +21 *Pf*Trx-eGFP and others (13), in which a non-positively charged cargo polypeptide is appended to, but structurally separated from, the PSC module, and internally grafted fusions, such as +21 *Pf*Trx-(HPV16-L2)_3x_, in which the non-PSC peptide module is embedded within the body of the PSC carrier protein. With respect to this feature, the uptake and intracellular (mainly cytosolic) distribution of +21 *Pf*Trx and its HPV16-L2 fusion derivative more closely resemble the internalization behavior of positively supercharged nanobodies (15), rather than that of +36 GFP fusions (8, 13). Importantly, both +21 *Pf*Trx-immune peptide epitope fusion derivatives and engineered PSC nanobodies lend themselves to direct (i.e., ‘terminal cargo-independent’) biomedical applications.

The high positive charge and Rcm values of +21 *Pf*Trx, which was primarily designed as a general carrier for peptide-antigen internalization, may allow to offset the potential negative charge contribution of the incorporated peptide (multi)epitopes. This would enable cell penetration of other peptide-antigens besides HPV16-L2(20-38)_3x_, but also peptide aptamers (e.g., peptides capable of interfering with specific protein-protein interactions), i.e., the very first bioactive peptide inserts for which thioredoxin was demonstrated to act as an effective scaffold and display protein (33, 34). On the other hand, considering the above-described Rcm vs. cell penetration relationship, a somewhat reduced internalization capacity might be expected for larger size peptide epitopes displayed on +21 *Pf*Trx. This might apply, for example, to the incorporation in the final antigen formulation of universal T-cell helper epitopes such as the PADRE peptide (35, 36), but also to more complex heterotypic HPV-L2 polytopes (37). Particularly interesting in this regard, would be genetic fusions with immunogenicity enhancers such as the c4-bp-derived OVX313 domain (57 aa), which self-assembles into an oligomeric structure composed by seven protomers each containing a cluster of five contiguous Arg residues (38, 39) and has previously been shown to accommodate genetically fused *Pf*Trx-displayed HPV-L2 polytopes (40). It is thus possible to imagine a synergistic effect on internalization (and immunogenicity) caused by seven +21 *Pf*Trx molecules exposed on the surface of the ring-shaped, positively charged OVX313 heptamer (overall theoretical Rcm value of 1.36/kDa). Such an effect would be consistent with the immunogenicity potentiation previously reported for other cell penetrating antigens (24, 25, 41), including artificial and viral capsid protein-derived (6, 26) nanoparticles. Also worth of note is the chemical compatibility of +21 *Pf*Trx-(HPV16-L2)_3x_ with the lipophilic AddaVax and the negatively charged CDA adjuvants, which did not interfere with, but rather slightly enhanced (especially CDA) PSC antigen internalization. Particularly interesting, in this regard, is CDA, whose internalization as a (meta)stable complex with a PSC antigen could favor interaction with the endoplasmic reticulum–resident receptor STING (42) (stimulator of interferon genes) and activation of a signaling pathway that induces the expression of interferon-β as well as multiple inflammatory cytokines. The reason(s) why a similarly favorable interaction was not observed for the CpG ODN adjuvant is presently unclear but worth of future more detailed investigations, also taking into account additional polyanionic adjuvants such as poly(I:C).

Considering the extensive resurfacing performed on a naturally evolved thermally hyperstable protein such as *P. furiosus* thioredoxin (13 substitutions on a total of 100 amino acid residues), the sizeable decrease in thermal stability observed with the +21 PSC variant is not so surprising. Especially, considering that most lysine substitutions (and possible repulsive effects) are located on loop elements that have previously been shown to represent key structural determinants of thioredoxin thermal stability (43). Less expected, was the approximately six-fold decrease in production yield of +21 *Pf*Trx compared to its wild-type counterpart. Given the still pretty high thermal stability of +21 *Pf*Trx and its (HPV16-L2)_3x_ insert-containing derivative (estimated Tm ~60°C), we do not believe that the observed drop in thermal stability is actually responsible for the reduced production yield. Another possible explanation, suggested by the need for freshly made transformants in order to achieve optimal expression yields, might be an increased bacterial cytotoxicity of the PSC protein. However, the fact that bacterial cell growth (OD_600_) as well as the wet-weight of induced, +21 *Pf*Trx-expressing cell pellets were not so different from those of bacteria expressing wt-*Pf*Trx (data not shown) argues against cytotoxicity as a major determinant of reduced production yield. We also note that adequate (although not top-ranking) production yields could be obtained with a standard expression *E. coli* strain [BL21 codon plus (DE3)], without the need to resort to special strains [e.g., BL21(DE3) pLysS] specifically designed for the expression of really cytotoxic proteins. Another, more likely possibility, is that the reduced production yield is due to the ‘electrostatic stickiness’ of +21 *Pf*Trx and its tendency to bind, and be lost in association with, a variety of cellular polyanions, especially nucleic acids. In fact, to achieve optimal production yields, bacterial lysis had to be performed in a high-salt (2 M NaCl-containing) medium, in order to favor dissociation from cellular polyanions and attain a normal chromatographic behavior of the +21 *Pf*Trx proteins.

Removal of MHC class II-recognized B- and T-cell epitopes through genetic resurfacing has been used to reduce the immunogenicity of therapeutic proteins (10). At first glance, the polycationic resurfacing of +21 *Pf*Trx might represent a disadvantage for this protein, which is intended to be used as an immune epitope-displaying scaffold. For example, we have previously reported the presence in wt-*Pf*Trx of an experimentally validated T-cell epitope [c3 (37)], into which three lysine substitutions have been introduced as a consequence of resurfacing. However, although we cannot exclude that this particular T-cell epitope might have been inactivated, new epitopes might have been concomitantly created. Indeed, an *in silico* analysis predicts a better T-cell immunogenicity score for the mutated compared to the wild-type c3 epitope (data not shown). The same analysis also predicts a consistently higher T-cell immunogenicity score of newly created mouse and human MHC class II recognizable T-cell epitopes in +21 *Pf*Trx compared to wt-*Pf*Trx. T-cell epitope reconfiguration mediated by polycationic resurfacing might thus add to the cell penetration capacity of +21 *Pf*Trx-(HPV16-L2)_3x_ in determining its overall enhanced immunogenicity that resulted in a sustained immune response even in the absence of an exogenously supplied adjuvant.

To our knowledge, all studies dealing with cell internalization-dependent immunogenicity potentiation reported so far relied on various kinds of CPP- or TLM-engineered macromolecular assemblies (6, 24–26, 41) as well as on synthetic nanocarriers (22), rather than on resurfaced positively charged proteins as immune-epitope carriers. In a different but PSC protein-related set-up, nanoparticles formed by the non-covalent assembly of +36 GFP and the HPV16 E7 oncoprotein were found to be capable of cell penetration and were reported to be significantly more immunogenic than the free E7 antigen (27). Compared to the latter nanoparticles, however, structural homogeneity, consistency of production and the lack of a specific requirement for a negatively charged cargo antigen represent clear advantages of a single-molecule +21 *Pf*Trx-based immunogen. Interestingly, nanoparticle design has been shown to influence the type of immune-response preferentially induced by the entrapped antigen (27). Although the impact of *Pf*Trx supercharging on cellular immunity remains to be determined, the faster immune response elicited by +21 *Pf*Trx-(HPV16-L2)_3x_, its self-adjuvancy, and the overall effect of the +21 *Pf*Trx scaffold on the induction of neutralizing anti-HPV16 antibodies appear to be remarkable and highly promising features of this newly designed antigen formulation. Furthermore, the enhanced immunogenicity observed upon intramuscular administration of the 21 *Pf*Trx-(HPV16-L2)_3x_ antigen suggests that its cell penetration capacity may apply to multiple tissues and cell types, likely including dendritic (antigen-presenting) cells, in addition to the HeLa cells we have utilized in this work for *in vitro* testing.

## Materials and methods

### Structure and electrostatic surface potential prediction

The structures of +21 *Pf*Trx and its derivatives were initially built with the Swiss Model 3D structure prediction Server (44), using the crystal structure of an ancestral thioredoxin (3ZIV, model identity 28.13%) as template (45). Further refined, high-confidence structures were subsequently generated with the recently released ColabFold implementation of the neural network-based, deep-learning modelling tool AlphaFold2 (32), using the following parameters: single sequence mode with MMseqs2 (Uniref+Environmental), model type auto, three recycles. The best +21 *Pf*Trx predicted structure featured a confidence, position pLDDT score value of 94.5 on a 0-100 scale. The corresponding pLDDT scores for +21 *Pf*Trx-eGFP and +21 *Pf*Trx-(HPV16-L2)_3x_ were 94.0 and 67.3, respectively. The lower score value of the +21 *Pf*Trx-(HPV16-L2)_3x_ structure reflects the low-confidence in the prediction of the three tandemly repeated L2 (aa 20-38) peptides displayed on thioredoxin. Electrostatic surface potentials were determined with the APBS software (46) and displayed with the ChimeraX Software (47).

### Recombinant +21 PfTrx protein and monoclonal antibody production

Codon-optimized sequences coding for the +21 *Pf*Trx, +21 *Pf*Trx-eGFP and +21 *Pf*Trx-(HPV16-L2)_3x_ polypeptides were chemically synthesized (Eurofins MWG Operon) and inserted into the *NdeI* site of a modified 6xHis-tag pET28 plasmid (Novagen). After sequence verification, the resulting constructs were transformed into *Escherichia coli* BL21 codon plus (DE3) cells for recombinant protein expression. Induction was performed by overnight culture (LB medium, 30°C) in auto-inducing medium (48), followed by cell harvesting and bacterial lysis by sonication (Misonix Sonicator 3000) in 25 mM Tris-HCl (pH 7.5), 2 M NaCl, plus one tablet of EDTA-free Complete Protease Inhibitor (Roche) per 50 ml buffer. After centrifugation (15,000 x g for 30 min at 4°C), the resulting soluble supernatant was immediately subjected to a two-step chromatographic fractionation procedure performed by metal-affinity (1 ml HiTrapCrude column, GE Healthcare) and cation exchange (5 ml MonoS column, GE Healthcare) chromatography. To this end, the supernatant fraction derived from a 1-liter bacterial culture (typically 50 ml) was first loaded onto a HiTrap Crude column pre-equilibrated in 25 mM Tris-HCl (pH 7.5), 20 mM imidazole, 2 M NaCl. After extensive washing, performed at a flow-rate of 1 ml/min, protein was eluted by applying a 20 ml linear gradient of the same buffer containing 500 mM imidazole. Pooled peak fractions, identified by SDS-polyacrylamide gel electrophoresis (SDS-PAGE), were exchanged into 25 mM MES (pH 6.5) by diafiltration and loaded onto a cation-exchange MonoS column equilibrated in the same buffer. Protein elution was performed at a flow-rate of 1 ml/min by applying a linear, 6-column volumes gradient of 0-2 M NaCl in 25 mM Tris-HCl (pH 7.5). +21 *Pf*Trx, +21 *Pf*Trx-(HPV16-L2)_3x_ and +21 *Pf*Trx-eGFP eluted around 1 M, 0.7 M and 0.6 M NaCl, respectively. Following SDS-PAGE analysis, individual peak fractions were pooled, exchanged into 25 mM Tris-HCl (pH 7.5), 0.3 M NaCl buffer, supplemented with the P8340 protease inhibitor cocktail (Sigma-Aldrich) and stored at -80°C. Depending on the column (see also below), an ÄKTA Prime Plus or an ÄKTA Pure 25M (GE Healthcare) chromatographic system was used for protein purification.

The purified +21 *Pf*Trx protein was used to immunize 6-8 month-old female Balb/c mice (Charles River, Sulzfeld, Germany). Following analysis of immune sera by ELISA using a GST-fusion derivative of +21 *Pf*Trx as capture reagent, mice with the strongest immune reactivity were screened for mAb production (49) using a bacterial lysate containing the unfused GST protein as a negative control. Positive hybridoma clones were identified by ELISA using the GST-fused +21 *Pf*Trx protein as capture reagent. After three rounds of subcloning using the same selection procedure, the hybridoma cell culture supernatant SK4E2 was selected and used for immunoblot and immunofluorescence analyses.

### Biochemical analyses

The native molecular weight of the +21 *Pf*Trx and +21 *Pf*Trx-(HPV16-L2)_3x_ proteins was determined by size exclusion chromatography (SEC) performed on an analytical Superose 6 Increase 5/150 GL column (GE Healthcare; 0.25 ml/min) equilibrated in 25 mM Tris-HCl (pH 7.5), 150 mM NaCl, using thyroglobulin (670 kDa), ovalbumin (45 kDa) and lysozyme (14.5 kDa) as molecular mass standards. A higher salt (2 M NaCl) buffer and a Superdex 200 5/150 analytical column (GE Healthcare) were used for +21 *Pf*Trx-eGFP.

Dynamic light-scattering (DLS) analysis, performed with a Zetasizer Nano ZSP apparatus (Malvern Instruments), was used to determine the hydrodynamic size of the proteins. Prior to DLS analysis, protein samples (0.5 mg/ml in 25 mM Tris-HCl, pH 8.0, 150 mM NaCl) were centrifuged (15,000 x g, for 15 min at 4°C) to remove any aggregate. DLS measurements were conducted at 25°C using 1 cm ZEN0040 disposable cuvettes and a 120 μl protein volume, with a measurement angle of 173° backscatter. The average hydrodynamic diameter of each protein was calculated based on three replicate measurements (60 s each).

Far-UV circular dichroism (CD) spectroscopic analysis (200–260 nm; average of 4 scans) and CD-assisted thermal unfolding studies (25-90°C range) were performed with a Jasco J715 Spectropolarimeter equipped with a Peltier temperature controller (0.2 cm path-length cuvette, bandwidth of 1 nm, data pitch of 0.5 nm, response time of 4 s) as described previously (28, 40).

### DNA binding studies

Complex formation between +21 *Pf*Trx and M13 DNA (8 kbp, 5.3x10^6^ Da) was initially investigated by Atomic Force Microscopy (AFM) using a previously described experimental set-up (40). Briefly, +21 *Pf*Trx or wt-*Pf*Trx as a control (1 µg each) were incubated for 15 min at 25°C with 200 ng of M13 plasmid DNA dissolved in 25 mM Tris-HCl (pH 9.0), 0.1 M NaCl in a final volume of 10 µl, then diluted 1:100 in 10 mM NaCl, 4 mM MgCl_2_, 4 mM HEPES (pH 7.4) prior to deposition onto freshly cleaved mica. This was followed by milliQ water washing, drying with a stream of nitrogen and imaging, performed in tapping mode in air with a Nanoscope IIIA microscope (Digital Instruments) equipped with an E scanner and a HQ : NSC14/Al BS tip (MikroMasch). Square images of 512_512 pixels were collected with a scan size of 1 μm and analyzed with the Gwyddion software (v2.45). The interaction between +21 *Pf*Trx and M13 DNA was also analyzed by electrophoretic mobility shift assays (EMSA) (50), which were conducted in the presence of a fixed amount (2 µg) of +21 or wt PfTrx and increasing amounts of M13 DNA (50-1000 ng), incubated for 15 min at 25°C in 25 mM Tris-HCl (pH 9.0), 0.1 M NaCl in a final volume of 20 µl prior to EMSA analysis.

SEC analysis was used to study the binding of a fixed amount (3 µg) of a single-stranded 64-mer oligonucleotide (21.1 kDa) to increasing amounts (up to 25 µg) of +21 or wt *Pf*Trx and to a fixed amount (30 µg) of hen egg lysozyme, that was used as a low-positive charge independent control. Samples were incubated for 15 min at 25°C in a final volume of 40 µl prior to SEC fractionation on a Superdex 75 5/150 analytical column (25 mM Tris-HCl, pH 7.5, 0.15 M NaCl), performed with an ÄKTA Pure 25M chromatographic system (flow-rate: 0.25 ml/min; detector wavelength: 280 nm).

### Cellular internalization studies

HeLa cells (ATCC, CCL-2) were cultured to 75% confluence at 37°C (5% CO_2_, 95% humidity) in Dulbecco’s modified Eagle’s medium (DMEM; Gibco-Thermo Fisher Scientific) supplemented with 10% fetal bovine serum (FBS; Gibco), 2 mM glutamine, 100 I.U. penicillin, 100 μg/mL streptomycin and 2.5 μg/mL of Amphotericin B. Individual supercharged proteins or the wt-*Pf*Trx control, pre-diluted in serum-free DMEM, were then added to the cells at the required concentration (specified in the text) and incubated for 4 h at either 37°C or 4°C as indicated.

To monitor +21 *Pf*Trx-eGFP uptake, cells were washed three times with heparin (20 U/ml) dissolved in phosphate-buffered saline (PBS), in order to remove non-specifically adsorbed protein and directly visualized by fluorescence microscopy (Axiovert S100; Zeiss); images were acquired and processed with the Axiovision (Rel 4.6) software.

For immuno-fluorescence imaging experiments monitoring the subcellular distribution of the internalized +21 *Pf*Trx proteins, HeLa cells were loaded in 12-well plates (~8x10^5^ cells/well) and after incubation and heparin washing as above, were fixed with 2% paraformaldehyde, followed by double-quenching with ammonium chloride in PBS and permeabilization with 0.2% (v/v) Triton X-100. After an additional washing with PBS and treatment with bovine serum albumin (BSA; 1% in PBS) in order to saturate non-specific binding sites, cells were incubated overnight at 4°C with 4′,6-diamidino-2-phenylindole (DAPI) and specific primary antibodies: anti +21 *Pf*Trx (non-diluted hybridoma cell culture supernatant of clone SK4E2); anti-wt-*Pf*Trx (51) diluted 1:50 in 1% BSA-PBS)**;** anti-Early Endosomal Antigen, EEA (diluted 1:200, Cell Signaling, USA); anti-lysosomal-associated membrane protein 1, LAMP-1 (diluted 1:200, Cell Signaling, USA). After washing with PBS, 1 hr. incubation at 37°C with the appropriate secondary antibodies (AlexaFluor 488-conjugated goat anti-rabbit IgG antibody and AlexaFluor 594-conjugated goat anti-mouse IgG antibody, Life Technologies, Carlsbad, USA) diluted 1:200 in 1% BSA-PBS, and coverslip mounting/sealing onto slides, cells were visualized using a Cell Observer microscope (Zeiss) and the resulting images were processed with the ImageJ software (NIH).

The same experimental set-up was used to assess the chemical compatibility with +21 *Pf*Trx-(HPV16-L2)_3x_ and effect on internalization of the AddaVax (*In vivo*Gen), aluminum hydroxide (*In vivo*Gen), aluminum phosphate (Sigma-Aldrich), CpG ODN (Sigma-Aldrich) and cyclic di-AMP (CDA; ASA Spezialenzyme GmbH, Wolfenbüttel, Germany) adjuvants. +21 *Pf*Trx-(HPV16-L2)_3x_ was preincubated at 25°C for 1h in the presence or in the absence the adjuvant before incubation for 4 h at 37°C with HeLa cells, at a final concentration of 0.5μM. Control experiments were conducted by incubating the adjuvant alone. In the preincubation samples AddaVax was 50% (v/v), cyclic di-AMP 7.5 µg, while several CpG/protein ratios were tested (from 1:5 to 1:50 w/w adjuvant/protein), but in all cases protein rapidly became insoluble. Aluminum hydroxide or aluminum phosphate were used in a 2.5:1 w/w adjuvant/protein ratio.

For cytotoxicity assays, confluent HeLa cells cultured in multiwell plates as described above, were incubated for 4 h in the presence of different concentrations (20 to 1.25 µM) of either wt- or +21 *Pf*Trx. Cell viability was measured after 24 h with the 3-(4,5-dimethylthiazol-2-yl)-2,5-diphenyltetrazoliumbromide (MTT) assay. Reduction of MTT by viable cells and formation of the purple-colored formazan product was determined by measuring absorbance at 540 nm (using 690 nm as reference wavelength) with a microplate reader. Four technical replicates were performed for each protein sample.

For immunoblotting analysis, following incubation for 1 hr. at 37°C or 4°C in the presence of +21 *Pf*Trx-(HPV16-L2)_3x_ as described above, HeLa cells were pelleted, the supernatant was removed, and cells were washed three times with heparin (20 U/ml) at 4°C. Cells were then collected by trypsinization, centrifuged, resuspended in 80 µl of RIPA lysis-buffer (50 mM Tris-HCl, pH 8.0, 150 mM NaCl, 1% Triton X-100, 0.1% SDS), kept on ice for 20 min and centrifuged at 220 x g for 15 min at 4°C. Protein concentration was determined with the Bradford assay (Bio-Rad) and 25 µg total protein equivalents of each supernatant were loaded onto 15% polyacrylamide gels and subjected to SDS-PAGE, followed by electro-transfer onto PVDF membranes (Bio-Rad). Blotted membranes were then incubated for 1 hr. in Tris-buffered saline containing 0.3% Tween-20 and 5% skim-milk, followed by 1 hr. incubation at 25°C with the anti-L2(20-38) mAb K4 (5, 28, 48) diluted 1:3000 in Tris-buffered saline lacking skim-milk. After washing with PBS, the membranes were incubated for 1 hr. with an IRDye 680-conjugated, goat anti-mouse secondary antibody (Li-Cor; diluted 1:15000). Following three additional washings with 50 mM Tris-buffered saline containing 0.3% Tween-20, immune-reactive bands were visualized by near infrared fluorescence using a ChemiDoc MP Imaging System (Bio-Rad). Quantitative data on endosomal/lysosomal markers colocalization, expressed as Pearson’s correlation coefficient values, were obtained by analysis of immunofluorescence microscopy images with the ImageJ suite software (52).

### Mouse immunization and pseudovirion-based neutralization assays

Six- to eight-weeks-old female BALB/c mice (Charles River; Sulzfeld, Germany; 5-10 animals/group) were immunized intramuscularly at biweekly intervals with 20 μg of the detoxified (28) and filter-sterilized +21 and wt *Pf*Trx-(HPV16-L2)_3x_ antigens. A standard (one priming-three boost injections) or a shorter (one priming-two boost injections) immunization protocol was employed, as indicated. AddaVax (50% v/v)- or CDA (7.5 µg/dose)-adjuvanted as well as unadjuvanted *Pf*Trx-(HPV16-L2)_3x_ antigens in PBS were delivered in a final volume of 100 µl. Blood samples were collected by cardiac puncture four weeks after the last immunization, followed by immune-sera recovery after a two-hours incubation at room temperature and centrifugation at 4500 rpm for 10 min.

Pseudovirion preparation and L1-PBNAs were performed as described (37, 40). Statistical significance of neutralization assay results and of the differences between the different vaccine treatment groups was determined with the non-parametric Mann-Whitney-Wilcoxon test performed with the GraphPad Prism software 5.00; differences between groups were considered significant at p < 0.05.

For IgG isotype profiling, Serocluster 96-well “U” bottom plates (Costar, USA) were coated with 0.2 µg/well streptavidin (Sigma-Aldrich, Germany) overnight at 37°C. On the next day, 0.03 µg of N-terminally biotinylated-HPV16 L2 peptide (GGSGKTCKQAGTCPPDIIPKVEGK) (GenScript Biotech, Netherlands) was added to the plates, which were incubated for 1 h at room temperature. Sera of mice immunized with the *Pf*Trx-(HPV16-L2)_3x_ and +21 *Pf*Trx-(HPV16-L2)_3x_ antigens were pooled separately, diluted at 1:100 in PBS containing 1.5% milk and 0.3% Tween 20, and added in duplicate to the L2 peptide-containing plates, which were then incubated for 1 h at 37°C. Anti-L2 antibody isotyping was performed with horse-radish-peroxidase (HRP)-conjugated goat-anti-mouse IgG1, IgG2a, IgG2b, IgG3, IgA and IgM (Southern Biotech, USA), following incubation for another 1h at 37°C. The colorimetric reaction was quantified at 405 nm with Multiskan Go (Thermo Fisher Scientific, USA) after 8 min. The K18 mouse monoclonal antibody (IgG1) was used as control.

## Data availability statement

Publicly available datasets were analyzed in this study. This data can be found here: PDB: 3ZIV, https://www.rcsb.org/structure/3ZIV.

## Ethics statement

Animal experimentation procedures were approved by the Regierungspräsidium Karlsruhe under permits A2/17 (SK4E2 hybridoma experiments) and G248/16 (immunization with vaccine prototypes), and were performed in accordance with the relevant guidelines and regulations. Mice were kept and handled in the animal house facility of the German Cancer Research Center (DKFZ, Heidelberg) under pathogen-free conditions, in compliance with the regulations of the Germany Animal Protection Law.

## Author contributions

DC, AB, and SO conceived the study. DC and GS, recombinant protein design, production and characterization. FM, FR, VF, and GD, immunofluorescence microscopy experiments. SM, atomic force microscopy and protein-DNA interaction experiments. FM and MM, immunization experiments and neutralization assays. DC, GS, GD, MM, AB, and SO, data analysis and critical revision of text and figures. AB, MM, and SO, fund retrieval. SO wrote the paper. All authors contributed to, reviewed and approved the final version of the paper.

## Funding

GS was partly supported by a post-doc fellowship from the Interuniversity Consortium for Biotechnology (CIB). FM was the recipient of a postdoctoral fellowship granted by the Baden-Württemberg Stiftung (Project number WSF-030). This work, which also benefited from the resources made available within the COMP-HUB Initiative (Dept. Chemistry, Life Sciences and Environmental Sustainability) funded by the “Departments of Excellence” program of the Italian Ministry for Education, University and Research (MIUR, 2018–2022), was supported by local funding (FIL 2018-21) granted by the University of Parma to AB and SO.

## Acknowledgments

We thank Valentina Garrapa (preclinics Italia S.r.l.) for initial hints on the potential interest of a PSC carrier protein for peptide antigen presentation and Elisabetta Levati (Department of Chemistry, Life Sciences & Environmental Sustainability, University of Parma) for assistance with recombinant protein expression.

## Conflict of interest

PSC PfTrx and +21 PfTrx-(HPV16-L2)3x as well as other thioredoxin derivatives are covered by patents US9303082B2 and US10736954B2, in which some of the authors of the present work (AB, GS, MM and SO) appear as co-inventors.

The remaining authors declare that the research was conducted in the absence of any commercial or financial relationships that could be construed as a potential conflict of interest

## Publisher’s note

All claims expressed in this article are solely those of the authors and do not necessarily represent those of their affiliated organizations, or those of the publisher, the editors and the reviewers. Any product that may be evaluated in this article, or claim that may be made by its manufacturer, is not guaranteed or endorsed by the publisher.
