# Supplementary material for: Enhanced immunogenicity of a positively supercharged archaeon thioredoxin scaffold as a cell-penetrating antigen carrier for peptide vaccines
Source: Front Immunol. 2022 Aug 9;13:958123. doi: 10.3389/fimmu.2022.958123 (PMC9405434; doi:10.3389/fimmu.2022.958123)
Supplement: Supplementary file 1 [file DataSheet_1.pdf]

Supplementary Figure 1

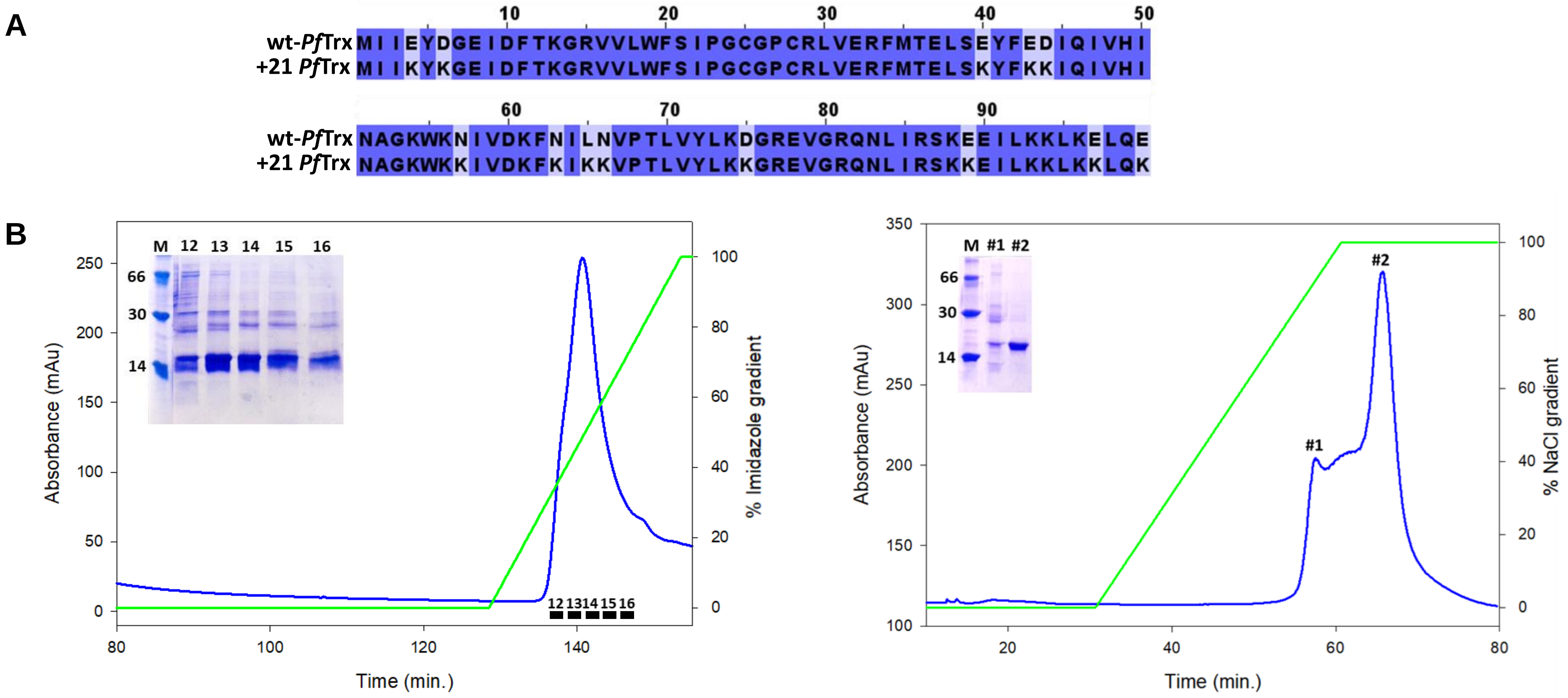

Supplementary Figure 1

C

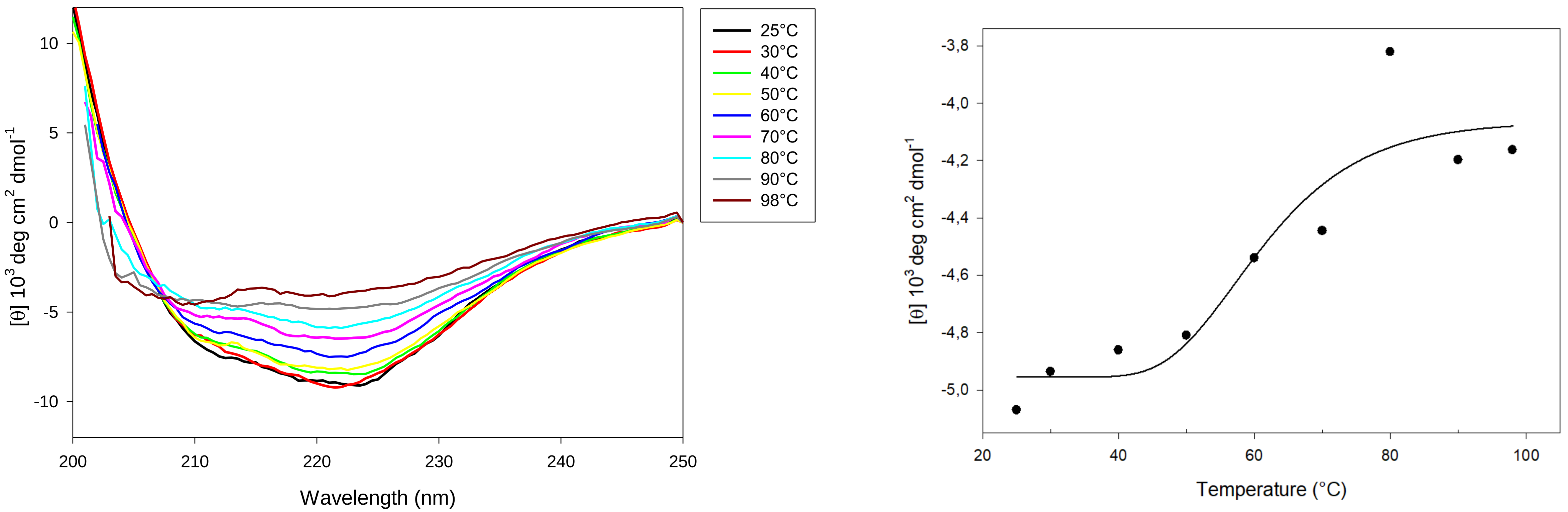

Supplementary Figure 1. Amino acid sequence, purification and thermal stability of +21 *Pf*Trx.

(A) Amino acid sequence of wt-*Pf*Trx (*upper sequence*) aligned with that of its positively supercharged, +21 *Pf*Trx derivative (*lower sequence*). *Pf*Trx amino acids replaced by lysine (K) residues in +21 *Pf*Trx are shown on a *light-blue* background. (B) Representative chromatograms of the metal-affinity (*left panel*) and the cation-exchange (*right panel*) chromatographic steps utilized for +21 *Pf*Trx purification. The SDS-PAGE profiles of the indicated chromatographic fractions are shown in the *insets*; fraction #2 in the *right-side panel* represents the homogenously purified +21 *Pf*Trx protein. Molecular mass markers (*M*; bovine serum albumin, carbonic anhydrase and lysozyme from top to bottom) are shown in the *left-side lane* of both *inset* images. (C) Thermal stability of +21 *Pf*Trx analyzed by circular dichroism. Far UV CD spectra were recorded from 200 to 250 nm at temperatures ranging from 25 to 98 °C (*left panel*); molar ellipticity values measured at 208 nm at the different temperatures are reported (*right panel*).

Supplementary Figure 2

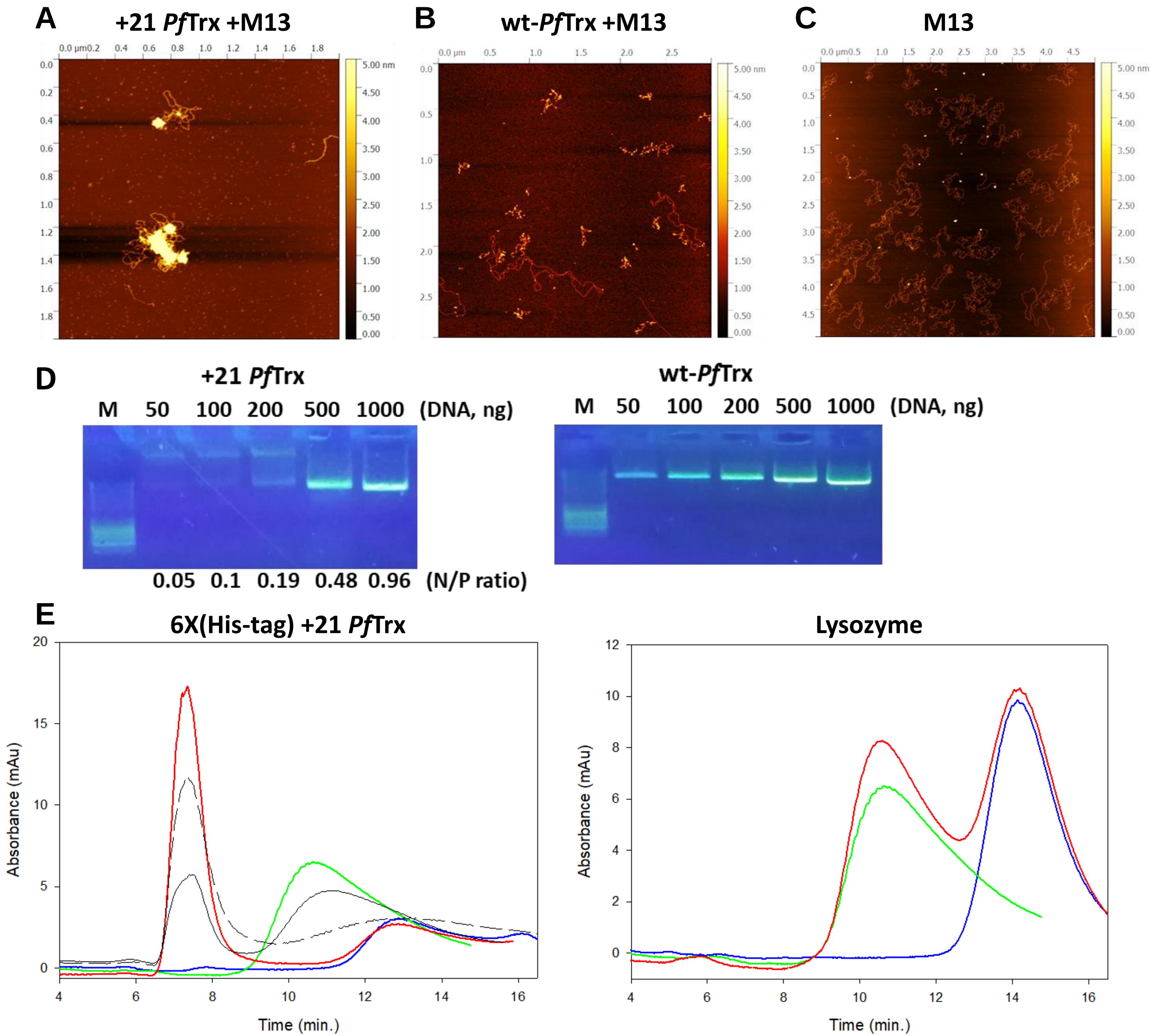

Supplementary Figure 2. DNA binding ability of +21 *Pf*Trx.

(A) Interaction of +21 *Pf*Trx with ds-M13 DNA (8 kbp, $5.3x10^6$ Da; 200 ng) visualized by Atomic Force Microscopy (AFM). The M13 DNA plus wt-*Pf*Trx and the M13 DNA only controls are shown in panels (B) and (C), respectively. The large nucleoprotein aggregates (200-500 nm in diameter) only observed upon incubation of M13 DNA with +21 *Pf*Trx (1 µg protein incubated with 200 ng of ds-M13 DNA for 15 min at 25°C in 25 mM Tris-HCl, pH 9.0, 0.1 M NaCl; same incubation conditions applied to the wt-*Pf*Trx control) are visible in the representative image shown in panel (A). (D) Interaction with M13 DNA analyzed by an electrophoretic mobility shift assay conducted in the presence of a fixed amount (2 µg) of +21 *Pf*Trx (*left-side panel*) or wt-*Pf*Trx (*right-side panel*) and the indicated amounts of M13 DNA under the same incubation conditions utilized for AFM analysis. Under the +21 *Pf*Trx panel, the DNA negative charges to protein positive charges ratio (N/P) for each assay is indicated**.** (E) Size Exclusion Chromatography (SEC) analysis (Superdex 75 analytical column, 25 mM Tris-HCl, pH 7.5, 0.25 ml/min) of the interaction between +21 *Pf*Trx and a single-stranded 64 nt DNA oligonucleotide. In the *left-side panel*, increasing amounts of +21 *Pf*Trx (6 µg, *solid line*; 10 µg, *dashed line*, 25 µg *red line*) were incubated with a fixed amount (3 µg) of the 64-mer oligonucleotide before SEC analysis. The protein concentration-dependent formation of an earlier eluting (*larger size*) macromolecular species, corresponding to the protein-DNA complex, is apparent; the *green*- and the *blue-line* chromatograms represent the DNA only and the protein-only controls, respectively. The results of an additional control performed with hen egg white lysozyme (30 µg), a protein similar in size to +21 *Pf*Trx but with a much smaller (+8) positive charge, and the 64-mer DNA oligonucleotide (3 µg) are shown in the *right-side panel*, where the *green*-, *blue*- and *red-line* chromatograms represent the DNA and lysozyme only controls and the DNA-protein mixture, respectively. At variance with the results obtained with +21 *Pf*Trx, no earlier eluting (*larger size*) macromolecular species corresponding to a protein-DNA complex was detected.

Supplementary Figure 3

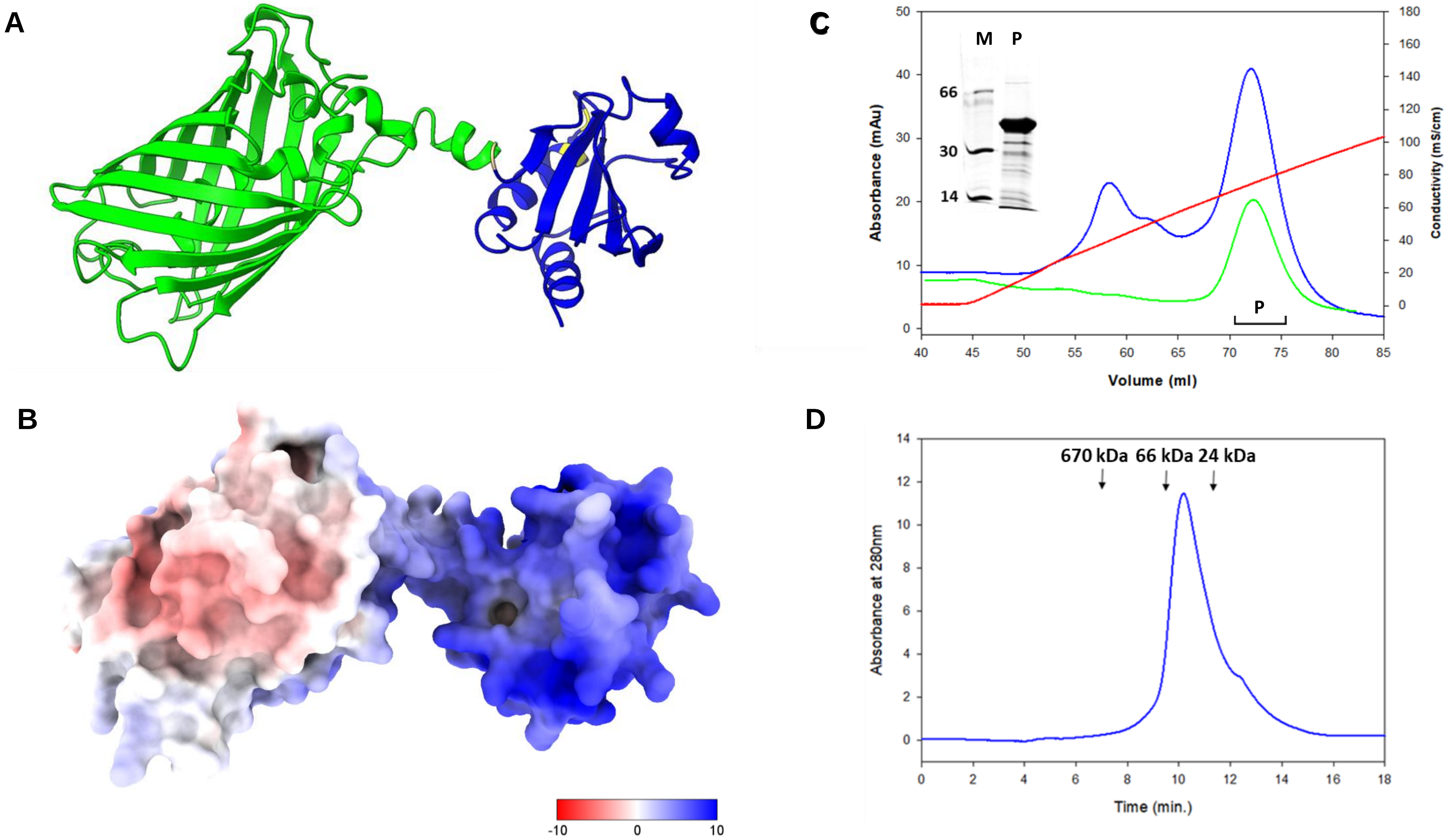

Supplementary Figure 3. Predicted structure and production of the +21 *Pf*Trx-eGFP fusion protein.

(A) 3D structure of +21 *Pf*Trx-eGFP predicted with AlphaFold2[45]; eGFP in *green* ribbon and +21 *Pf*Trx shown as a *blue* ribbon, with the Trx active/display sites Cys residues in *yellow*. (B) Electrostatic surface potential of the +21 *Pf*Trx-eGFP fusion protein represented in a false-color scale ranging from −10kT/e (*red*) to +10kT/e (*blue*). (C) After an initial metal-affinity purification step (*not shown*), +21 *Pf*Trx-eGFP was further purified on a MonoS cation exchange column (see ‘Methods’ for details). Protein elution was monitored both at 280 nm (*blue line*) and at 488nm (*green line*), and a pool of fractions (*P*) containing the purified protein was further analyzed by SDS-PAGE (*inset; M:* molecular mass markers, same as specified in Figure S1 legend; *P*: pooled chromatographic fractions loaded on the gel). (D) Native molecular weight of the +21 *Pf*Trx-eGFP fusion protein determined by SEC analysis performed on a Superdex 200 GL 5/150 analytical column.

Supplementary Figure 4

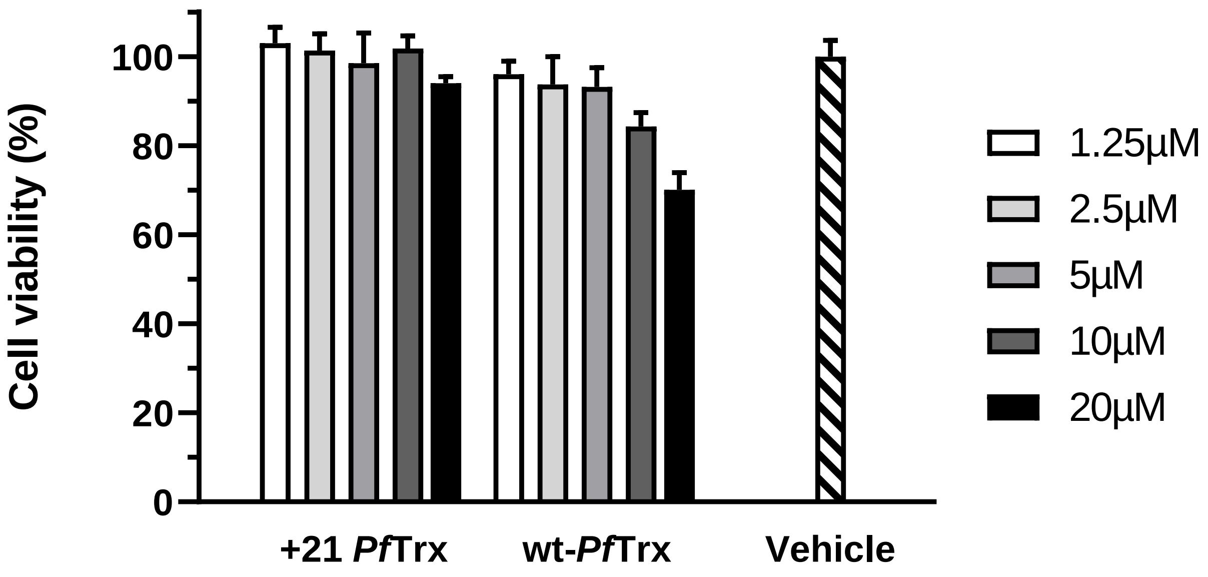

Supplementary Figure 4. Cytotoxicity assays.

Viability assays were performed with the MTT dye on HeLa cells treated for 4 h with the indicated concentrations of +21 *Pf*Trx (see 'Materials and Methods' for details); wt-*Pf*Trx served as a reference, non-PSC protein. Data, expressed as percent survival relative to a 'vehicle only' control, were collected 24 h after treatment and represent the mean ±SD of four technical replicates.

Supplementary Figure 5

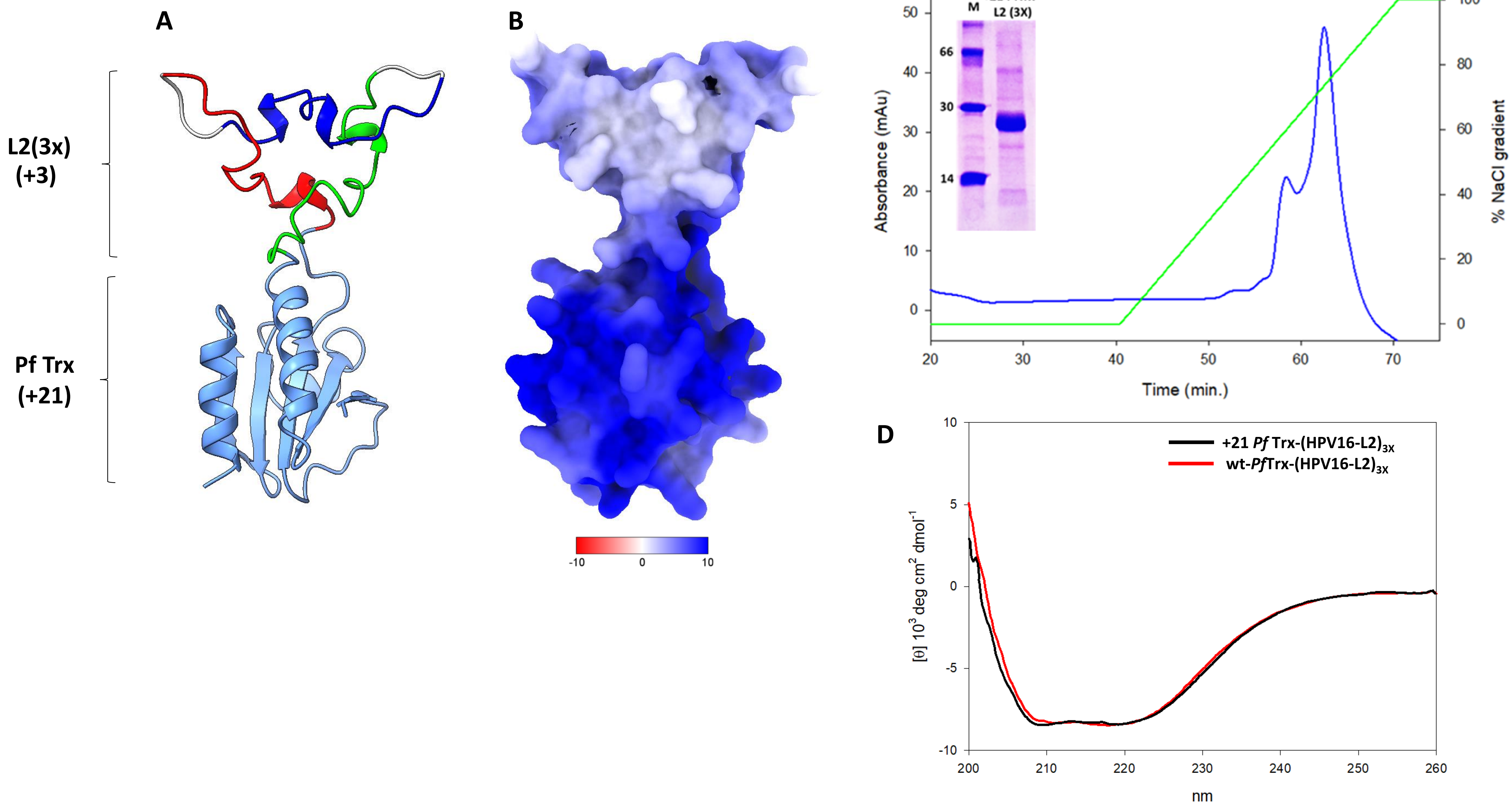

Supplementary Figure 5

E

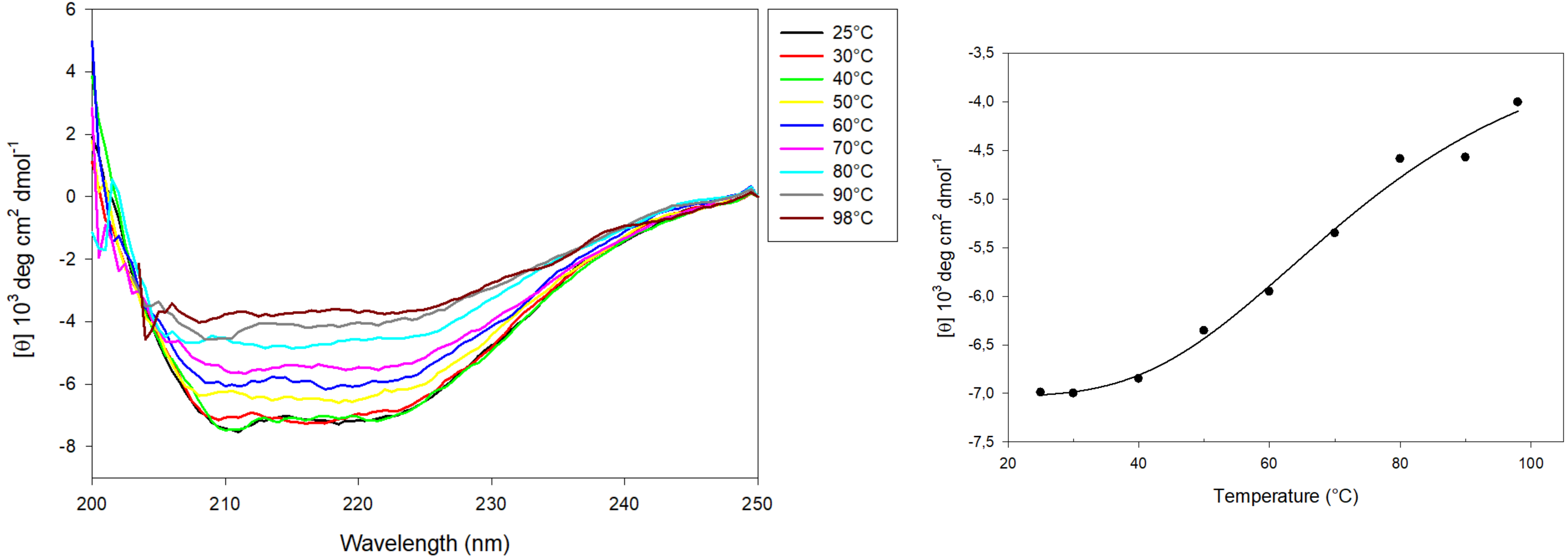

Supplementary Figure 5. Construction, predicted structure, purification and thermal stability of the +21 *Pf*Trx-(HPV16-L2)$_{3x}$ antigen.
(A) 3D structure of the +21 *Pf*Trx-(HPV16-L2)$_{3x}$ fusion protein predicted with AlphaFold2[45]; the +21 *Pf*Trx structure is shown as a *light-blue* ribbon with the three HPV16-L2 epitopes in *red*, *blue* and *green*. The overall net charge of the protein is +25 because of the (+3) and the (+1) charge contributions of the (HPV16-L2)$_{3x}$ peptide epitopes and the 6X His-tag linker peptide (not shown), respectively. (B) Electrostatic surface potential of the +21 *Pf*Trx-(HPV16-L2)$_{3x}$ protein represented in a false-color scale ranging from −10kT/e (*red*) to +10kT/e (*blue*). (C) MonoS cation exchange chromatography purification step of +21 PfTrx-(HPV16-L2)$_{3x}$; the pool of fractions (*P*) containing the purified +21 *Pf*Trx-(HPV16-L2)$_{3x}$ protein that was subjected to SDS-PAGE analysis and used for immunization experiments is indicated (*inset; M:* molecular mass markers, same as specified in Figure S1 legend; *P*: pooled chromatographic fractions loaded on the gel). (D) Comparative circular dichroism spectra of +21 *Pf*Trx-(HPV16-L2)$_{3x}$ (*black line*) and wt-*Pf*Trx-(HPV16-L2)$_{3x}$ (*red line*). (E) Thermal stability of +21 *Pf*Trx-(HPV16-L2)$_{3x}$ analyzed by circular dichroism. Far UV CD spectra were recorded from 200 to 250 nm at temperatures ranging from 25 to 98 °C (*left panel*); Molar ellipticity values measured at 208 nm at the different temperatures are reported (*right panel*).

Supplementary Figure 6

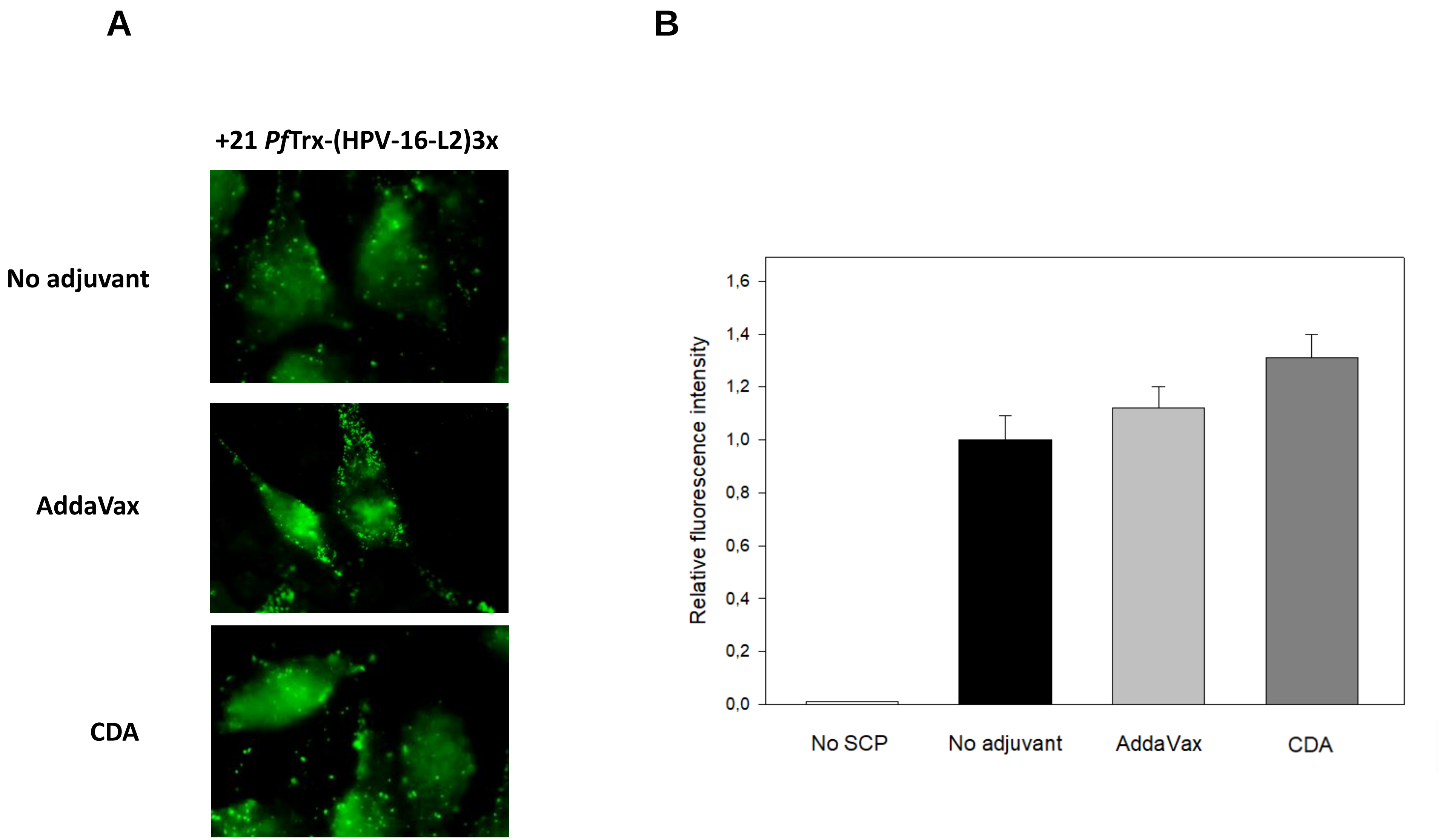

Supplementary Figure 6. Effect of adjuvants on PSC antigen internalization.

(A) Representative immunofluorescence microscopy images showing cellular internalization of +21 *Pf*Trx-(HPV-16-L2)$_{3x}$ without adjuvant or supplemented with the AddaVax or CDA adjuvants as indicated. No fluorescence signal was detected in control experiments performed with HeLa cells incubated with adjuvants alone (not shown). (B) Quantification of +21 *Pf*Trx-(HPV-16-L2)$_{3x}$ (+/- adjuvant) internalization data. The "*No SCP*" bar represents the negligible fluorescence measured in HeLa cells incubated with AddaVax alone (identical value has been measured for CDA, not shown); data, obtained with the ImageJ suite software, are the mean + SEM of three independent experiments.

Supplementary Figure 7

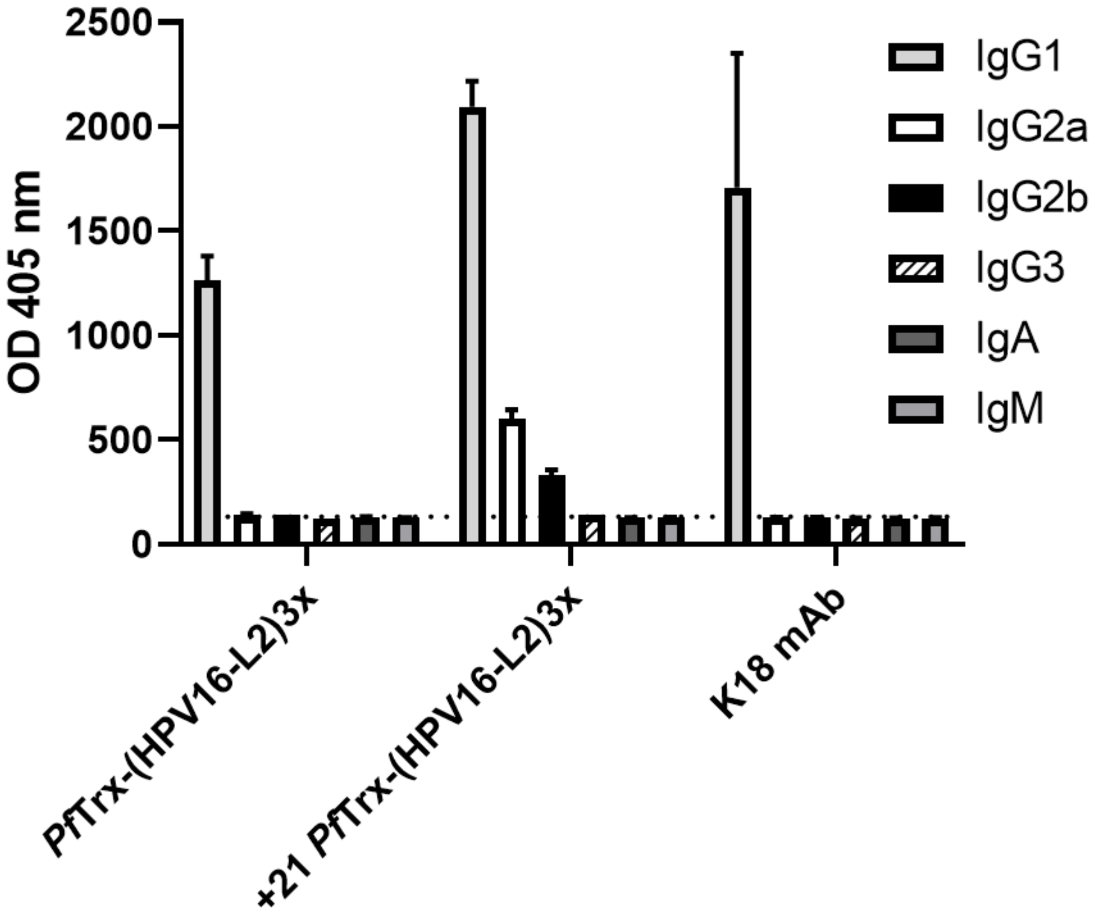

Supplementary Figure 7. Immunoglobulin isotype profiling of anti-HPV16-L2 antibodies.

Anti-HPV16 L2-specific immunoglobulin isotype distribution determined by antibody isotype ELISA (see 'Materials and Methods' for details). Measurements were performed on pooled immune-sera from mice immunized with the wt-*Pf*Trx-(HPV16-L2)$_{3x}$ or the +21 *Pf*Trx-(HPV16-L2)$_{3x}$ antigen. The mouse monoclonal anti-L2 antibody K18 (IgG1) served as a control for this analysis. Values are the mean ±SD of two technical replicates.
